# Global Health Education and Advocacy: Using BMJ Case Reports to Tackle the Social Determinants of Health

**DOI:** 10.3389/fpubh.2018.00114

**Published:** 2018-05-07

**Authors:** Nathan T. Douthit, Seema Biswas

**Affiliations:** ^1^BMJ Case Reports, London, United Kingdom; ^2^Graduate Medical Education, Brookwood Baptist Health, Birmingham, AL, United States

**Keywords:** global health, education, social determinants, case reports, advocacy

## Abstract

Since 2013, BMJ Case Reports (http://casereports.bmj.com/) has published over 70 global health case reports from five continents, written by doctors, nurses, students, and allied health professionals. These cases, a burgeoning repository of evidence of how real patients are affected by disease, trauma, violence, sexual assault, conflict, migration, adverse living and working conditions, and poor access to health care, discuss, in addition to clinicopathological findings, the global health problems affecting each patient. The global health problem analysis examines the problems of individual patients, critically appraises the literature, and describes actual and potential solutions for the patient, the local community, and patients affected by similar issues across the world. At present global health literature and learning materials lack a patient focus and real-life context in the analysis of global health problems. BMJ Case Reports global health case reports are a unique and important tool to learn about and advocate for change in the social, political, cultural, and financial determinants of health as they affect real patients. This growing evidence base brings together clinicians, local service providers, policy makers, and government and non-governmental institutions to effect real change in patients’ lives toward improving health. Each global health case report is an excellent resource for learning, and together, these case reports provide essential reading for anyone embarking on a career in global health, and writing their own case report. The online course (http://casereports.bmj.com/site/misc/GHMA_Mar_2017.pptx) at BMJ Case Reports uses these cases and is free to access.

## Introduction

Imagine a typical chronically ill patient who sees his doctor half an hour every three months. These four encounters each year—the physician’s opportunity to counsel, diagnose, and treat—constitute only 0.02% of the patient’s life. For all the tests—the 99.98% of the time that the patient is elsewhere, making decisions about his health in the context of his culture, family, and community—the doctor’s impact on the patient’s choices is minimal. (1)

This article outlines how health professionals may gain deeper insight into their patients’ lives, learn more about the patients’ families and communities, and learn how they are affected by disease and the real causes of disease—the social determinants of health. For health professionals to learn global health, they must become familiar with the stories of individual patients and populations; ideally sharing the same community and experiencing the health pressures of our patients. While there is an abundance of theory taught in global health, health professionals must be agents of change in the determinants of health and disease; health professionals must be moved to action. Calls to action to tackle the determinants of health have paralleled progress in medical science and have come from pioneers in medicine from Hippocrates to Hodgkin and Virchow to Marmot.

[Recommendations for global health curriculum] encouraged the honing of trainees’ skills by emphasizing, in addition to critical thinking, actual doing—either experientially, as in collaborations with outside organizations, or *through case studies and problem-based course work that mimics real-world content*. [(2), emphasis ours]

Physicians require a repository of evidence in their action against health inequalities and disparities in access to health care. BMJ Case Reports’ global health case reports form such a repository, providing real evidence of the need for change, a resource for teaching students and clinicians about the real causes of disease—the social determinants of health, and evidence of how real individuals are affected by public and global health issues—individuals living with disease and disability. There are other global health publications that serve as useful learning resources: Case Studies in Global Health (3) describe the work and impact of projects around the world; Global Health Reports (4) describe priority health issues, the case collection of the Global Health Delivery Project (5) provides teaching cases on global health issues and infectious diseases; and Case Reports in the Lancet describe important and interesting cases that complement articles in Lancet Global Health. If health professionals are to effect meaningful change in their patients’ health and lifestyles, however, knowledge of how individuals are affected by disease is invaluable. BMJ Case Reports global health case reports describe how individuals are affected by disease and the real causes of ill health. In dealing with disease professionals must address these determinants of ill health by helping their patients to make better choices.

It is the responsibility of a global health provider to practise health or humanitarian diplomacy by advocating for better standards of living and work, better access to health care, and equity in health-care service provision (6). The International Federation of the Red Cross defines this as: “Humanitarian diplomacy is persuading decision makers and opinion leaders to act, at all times, in the interests of vulnerable people, and with full respect for fundamental humanitarian principles” (7). For the health practitioners in action, the patient is at the center of all diplomatic efforts. These efforts require dialog and coordination with multiple partners—medical and non-medical, but in an increasingly specialized and fragmented modern medical environment, the treating physician needs to take the lead in the interdisciplinary approach to improving health and improving access to quality health care. Humanitarian diplomacy requires research into the social, cultural, environmental, financial, and geopolitical determinants of health, as well as responsibility for major health service policy and provider decisions, too often seemingly beyond the leverage of the individual patient and clinician. Diplomacy and advocacy are formidable tasks to add to the work of clinicians, however. There is little incentive for a clinician to invest the time and effort required for meaningful humanitarian diplomacy for a patient he or she hardly knows. In reading, and, more importantly, writing, global health case reports, the effects of social determinants and lofty theoretical definitions are made real, practical, and relevant. The better we know our patient, the more aware we are of their needs and the greater our motivation to see their lives improved. We care more. Caring moves us to action.

## Background and Definitions

### What Is “Global” Health?

“Global health” has been defined in many different ways. It is “worldwide improvement of health, reduction of disparities, and protection against global threats that disregard national borders” (8). The UK government sees global health as, “Health issues where the determinants circumvent, undermine or are oblivious to the territorial boundaries of states, and are thus beyond the capacity of individual countries to address through domestic institutions” (9). The ideas of transnational problems and health disparities are helpfully combined in the definition by Koplan et al.:

[A]n area for study, research, and practice that places a priority on improving health and achieving equity in health for all people worldwide. Global health emphasizes transnational health issues, determinants, and solutions; involves many disciplines within and beyond the health sciences and promotes interdisciplinary collaboration; and is a synthesis of population-based prevention with individual-level clinical care. (10)

**Figure 1:**
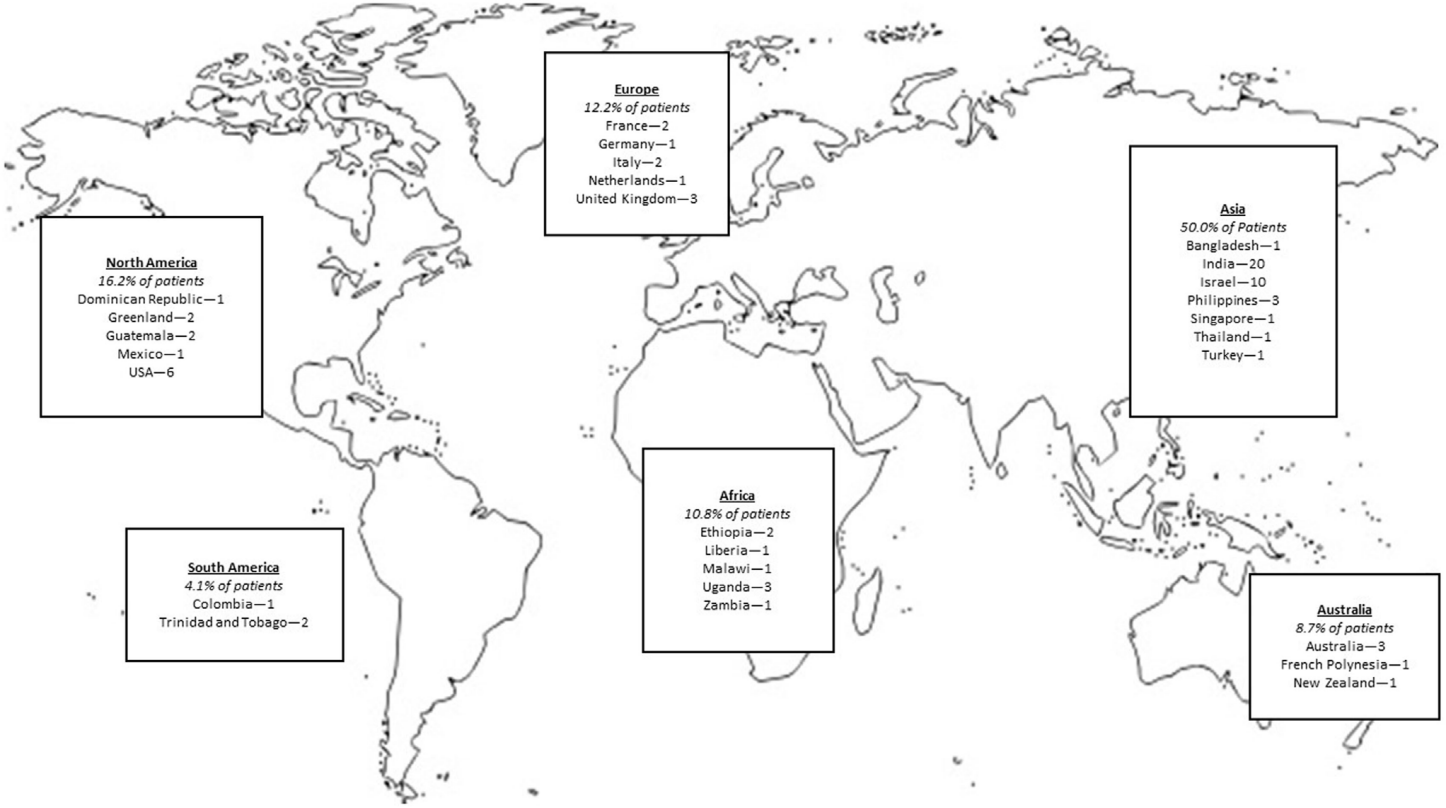
Patient locations in BMJ Case Reports global health cases.

This broad definition is helpful because it asserts that global health is not merely a study of pathology affecting patients in middle- and low-income countries. It involves social determinants of health, interdisciplinary communication and practice and holistic care for all patients, wherever they are (Figure 1). Global health is a study of what causes patients to be ill around the world, the social determinants of health. “[T]he quality of governance, the distribution of political power, and allocation of resources are all direct contributors to population health; they always have been and always will be” (11).

The complexity of global health problems demands creative interdisciplinary communication and practice to understand and implement effective and practical solutions. “Complex health needs of patients living in low-income communities require interventions that are innovative, multifaceted, and are drawn from the expertise of interprofessional teams in partnership with local communities” (12). Without collaboration, improved global health outcomes are unattainable. “Global health is a collaborative field that demands highly trained practitioners who can work across disciplines to achieve the innovations necessary to address complex, often intractable, public health concerns” (13).

It is only by changing factors that create and exacerbate illness that doctors can work with patients toward complete physical, mental, and social well-being and not merely the absence of disease and infirmity (14). Holistic care is integral to global health.

Integrated people-centered health services means putting people and communities, not diseases, at the center of health systems, and empowering people to take charge of their own health rather than being passive recipients of services. Evidence shows that health systems oriented around the needs of people and communities are more effective, cost less, improve health literacy and patient engagement, and are better prepared to respond to health crises. (15)

Even if a doctor never leaves his or her home town, in dealing with all the problems his or her patients face holistically, engaging with health and non-health professionals to effect solutions and in addressing diverse determinants of health and disease from political or financial to social or cultural, he or she is practising “global health.” This differs from public health in that an individual patient remains the focus of care and action. Taking on the global problems of hunger, poverty or health disparities is prohibitively difficult but finding lasting solutions to these problems as they affect *an individual patient or family* is not only feasible but integral to the practice of clinical medicine. Why send patients back to the same conditions that have made them ill in the first place? (16). Global health practitioners can most impact their patients’ behavior by using an interdisciplinary approach to address the patient as a whole person, and by fighting to change the social determinants that affect their patients. Marmot views this not as a choice for health-care professionals, but a responsibility:

Who will be the agents to bring to the attention of policy makers the need for such actions on the social determinants of health? Why not the medical profession? Who cares more about the tragedy of lives blighted by premature ill health than do we in the medical profession? If we care, we should be leading the charge for action across a broad front to reduce inequalities in health. (6)

**Figure 2:**
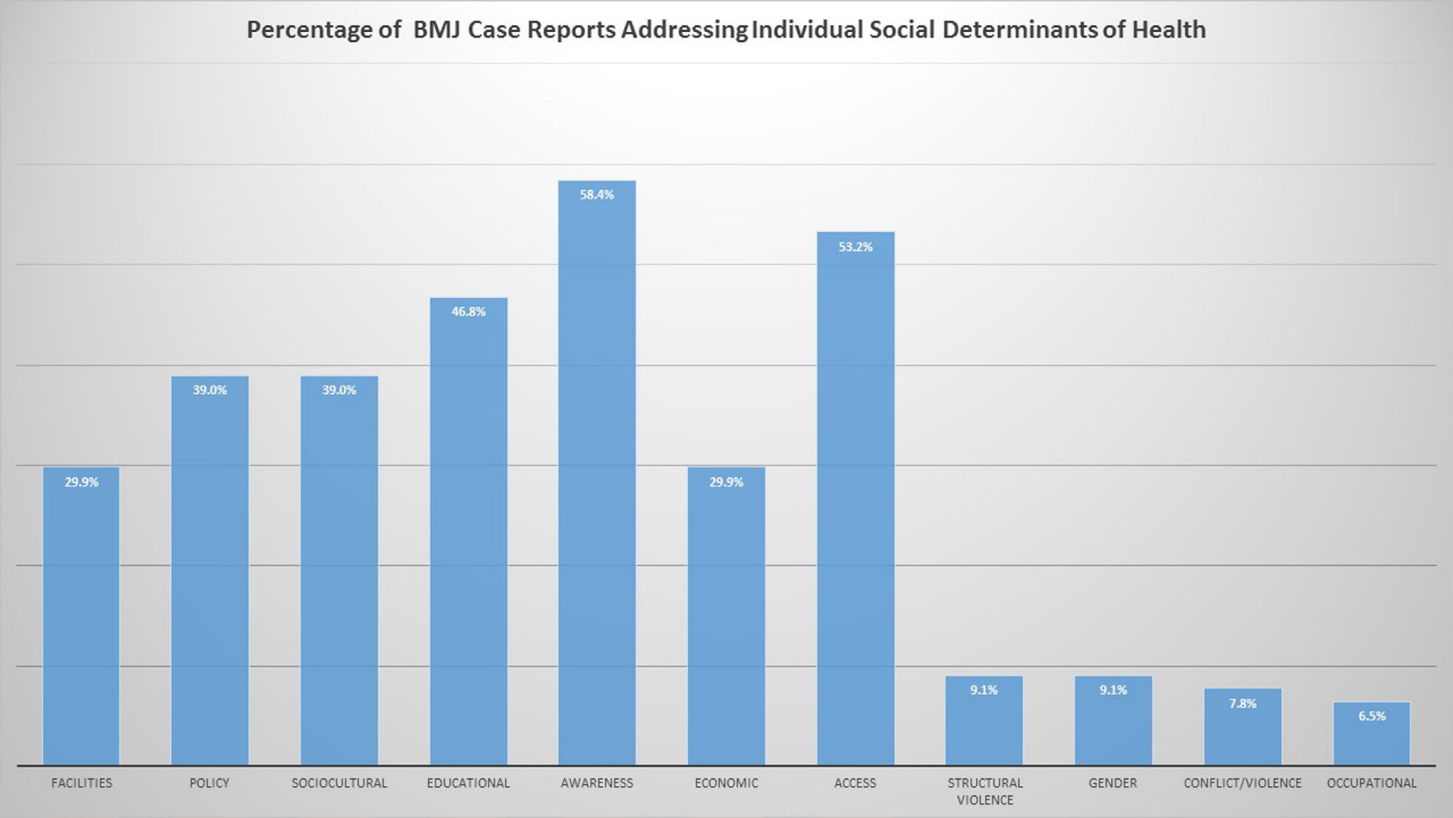
Social determinants of health addressed in BMJ Case Reports global health cases.

To train global health providers, case reports are uniquely situated to tackle social determinants of health. While it is neither wise nor ethical for students to practise “medical volunteerism,” students can experience the real-world implications of social determinants and global health by reading the global health case reports at BMJ Case Reports^1^ (17). Case reports have long been recognized as a useful tool for teaching health professionals and are a necessary component of any training in global health. (18, 19). There are many universities that use them extensively in global health training. (5, 20–22). BMJ Case Reports global health case reports and teaching resources provide a unique repository of teaching cases to describe and evaluate global health problems and to attain global health competencies (23). The cases focus on various social determinants of health, as can be seen in Figure 2.

### Practical Examples of Theoretical Definitions in Global Health

#### Social Determinants of Health

The environments in which humans are “born, grow, live, work, and age” have profound implications for their health (24). These are the social determinants of health. They include factors as intrinsic as gender, and as extrinsic as the geopolitical stability of their country and are shaped by politics, history, and wealth (10). According to the Director General of the World Health Organization (WHO) in 2013, “The challenges facing public health, and the broader world context in which we struggle, have become too numerous and too complex for a business-as-usual approach” (25).

The effect of the social determinants of health on individual patients is well illustrated in a case report by Douthit and Alemu (26). The case is of a young woman with nasopharyngeal carcinoma. The patient’s illness is inadequately evaluated and diagnosed as a consequence of the family’s poverty prohibiting access to quality health care. As her disease progresses, she is unable to work and contribute toward her family’s farm. Both she and her sister are forced to halt their education to manage her health. Thus, a cycle of economic deprivation continues. Poverty, lack of education and lack of economic opportunity were the key determinants of this patient’s health.

#### Structural Violence

Structural violence against individuals and communities creates ill health (27). Structural violence may be defined as follows:

[S]ocial arrangements that put individuals and populations in harm’s way …. The arrangements are structural because they are embedded in the political and economic organization of our social world; they are violent because they cause injury to people (typically, not those responsible for perpetuating such inequalities). With few exceptions, clinicians are not trained to understand such social forces, nor are we trained to alter them. Yet it has long been clear that many medical and public health interventions will fail if we are unable to understand the social determinants of disease. (28)

These effects are described in a case report by Borick writing about the life of a “Jogini” in India (29). As a result of the restrictive effects of the caste system limiting her family’s opportunity for economic development and her own educational development, the patient is made a ritual sex worker at the age of menarche. Her repeated abuse is sanctioned by society and eventually leads to her infection with HIV. The patient is able to seek help from a non-governmental organization (NGO), and cannot resume her life as a Jogini due to the risks this poses to her health and the community. She finds an advocate for her health in the NGO that assists her in leaving the cycle of structural violence that has resulted in abuse and HIV.

#### Vulnerability

“Across history, culture, and nation, ill persons are vulnerable, dependent, nervous, fearful and exploitable” (30). Vulnerability is created by many different factors, however, “Poverty is the single most important factor in determining vulnerability” (31). Race, status, and education play key roles (32). Regardless of their present physical health, vulnerable populations are only ever one misfortune away from catastrophe. They need resilient systems that can provide holistic care even in the midst of disaster.

Undocumented immigrants present a uniquely vulnerable population. Colavita et al. describe the case of a Gambian immigrant who entered Italy illegally (33). He had been forced to work in agriculture under the influence of an unknown drug, and to escape had been immobilized in the squatting position among other migrants on a boat for 3 days. He presented with severe rhabdomyolysis and his life at risk. Ensuring that the migrants have safe access to adequate health care after their arrival is the responsibility of the nations who receive them.

#### Disasters, Hazards, and Crises

According to Didier Cherpital, former secretary general of the International Federation of the Red Cross, “Disasters seek out the poor and ensure that they stay poor” (34). The effects of disasters both personal and corporate, natural and man-made conspire to ensure that the vulnerable remain ill (35). Disasters come from hazards, which are

a dangerous phenomenon, substance or human activity resulting in the loss of life or injury, damage to property, the loss of livelihood and public services, social and economic disruption, or environmental damage. (36)

These hazards are almost always man-made through population displacement, famine, and conflict. Disasters lead to humanitarian crises. A crisis is any event that threatens a large community and overwhelms capacity to respond.

Conflict prevents access to essential health-care delivery and increases the vulnerability of patients to injury, infectious disease and the complications of chronic disease. The effects of conflict are explored by Hayari et al. in a case report about surgical and oncological care delivered to a young woman from a war-torn country (37). The infrastructure of her country is destroyed by conflict. Surgical treatment of a dysgerminoma is complicated by lack of resources in a decimated health system. She seeks treatment in a neighboring country, returns home, and is potentially lost to follow-up. The capacity to provide good quality long-term care is lost as a result of conflict.

#### Resilience

Vulnerable populations need resilient societies for their protection. Resilience may be defined as, “the capacity for populations to endure, adapt and generate new ways of thinking and functioning in the context of change, uncertainty or adversity” (38). Resilience has also been defined as “[T]he capacity of health actors, institutions, and populations to prepare for and effectively respond to crises; maintain core functions when a crisis hits; and, informed by lessons learned during the crisis, reorganize if conditions require it” (39). Creating a resilient society and health system ensures that the most vulnerable will be protected in the midst of personal and population disasters. Success in building these systems can be gaged by health equity. The WHO has said

The development of a society, rich or poor, can be judged by the quality of its population’s health, how fairly health is distributed across the social spectrum and the degree of protection provided from disadvantage as a result of ill-health. (16)

In India, one case series makes clear that growing civilian violence and ease of access to firearms are a public health issue (40). The authors compare India’s trauma management to the UK’s, and advocate for development of an emergency medical services system, finding ways to improve the quality of trauma services at rural and urban centers, and innovative policy to retain expertise within the country’s workforce. They also advocate for greater government intervention to reduce civilian violence and access to firearms. These are the necessary steps to create a society and health-care infrastructure resilient enough to withstand the increase of patients with penetrating chest trauma.

#### Human Rights

Human rights are guarantees to individuals and populations against actions, either of omission or commission, that inhibit or risk universal freedoms and entitlements. They include “the right to life, liberty and security of person …. The right to the highest attainable standard of health …. The right to just and favorable working conditions. The right to adequate food, housing and social security. The right to education” (41).

Muralidhar describes the case of a man killed as a result of an occupational injury (42). Medical staff played an important role in assisting the man’s family in asserting their rights to learn the circumstances of his death. The Occupational Health and Safety Centre of Mumbai was able to advocate for justice for the man and his family and help ensure that this tragedy is not repeated.

#### Patient Rights

Patients are also entitled to specific rights regarding their health care. According to the WHO:

Freedoms include the right to control one’s health and body … and to be free from interference …. [These rights] include the right to a system of health protection that gives everyone an equal opportunity to enjoy the highest attainable level of health. (43)

Dunton et al. describe the case of a Bedouin patient in Southern Israel and his access of health-care services (44). Despite a robust universal health-care system and a culturally competent doctor, the patient still presented late in the course of his disease and was non-adherent to some aspects of his therapy. The stigma of his disease, the urbanization, and modernization of his society and cultural and societal factors prevented the patient from enjoying his highest possible level of health.

## Promoting Understanding to Change Behavior among Clinicians and Their Patients

Health-care professionals have an important role to play in upholding human and patient rights. In reading global health case reports at BMJ Case Reports, clinicians come face-to-face with the daily realities of living with disease for the most vulnerable. This study of the lives of individual patients promotes an understanding of the factors that affect their health and their lives. This understanding potentially leads to a change in their behavior and allows them to more effectively impact their patients’ behavior.

### Changing the Behavior of Global Health Providers

To be effective for their patients, health professionals must be culturally competent. A review by the National Center for Cultural Competence at Georgetown University found that culturally competent health professionals can decrease HBA1C percentages, increase uptake of cancer screening, reduce smoking, blood pressure, cholesterol, and BMI as well as improve nutrition (45).

Cultural competency goes beyond cultural awareness or sensitivity. It includes not only possession of cultural knowledge and respect for different cultural perspectives but also having skills and being able to use them effectively in cross-cultural situations. (46)

To make effective change, health practitioners need not be a part of the culture of their patients, but they must be trained to put aside their own ethnocentrism and integrate patients’ cultural beliefs into their health-care practices. If cultural factors are neglected, opportunities for health improvement will be missed as, “Much of what is clinically possible is set by these factors” (47). “Careful and appropriate implementation of sound cultural competency techniques in delivering health services could go a long way toward reducing disparities” (46).

Cultural competence springs from understanding the microenvironments and macroenvironments in which patient behavior takes place. “[For example, alcohol] consumption and patterns of behavior associated with consumption are highly variegated across different social groups. So what happens at a Jewish wedding, in a student union bar in Freshers’ week, in an English country pub on a quiet Tuesday lunchtime, at an Oxford college High Table, at a club for young people on a Saturday evening, on a cruise ship providing holidays for gay and lesbian people, at a middle class dinner party, in a town center pub where a group of middle-aged male manual workers are drinking after work, will all be different …. All have immediate preceding conditions and all have more distant preceding conditions and of course individual people may, and indeed are very likely to inhabit several, and many other situations on different occasions in their lives” (48).

Without an understanding of the patterns of behavior influenced by the environments in which the patient lives there can be no hope of meaningful behavioral change. We are reminded that, “The so-called chain of logic has many broken links.” “[K]nowing one’s audience is critical. Being aware of all the influences on the current state of affairs will help create realistic expectations about how much change is possible and the barriers to address” (49). It has been shown that dynamics of alcohol use and risky sexual behaviors, while following some general trends, are different according to the local settings and cultures in which they take place. “[S]ocial dynamics of alcohol use and sexual risk behaviours warrant a search for culture-specific and context-specific ways of dealing with the problem.” In global health, “The importance of a public health preventive emphasis has been underlined, i.e. a concern with individuals as well as the environment (settings) within which these individuals find themselves” (50). Smoking patterns have also been shown to be culture specific (51). The environment of health decisions and culture of different populations must be understood by health professionals. Once this understanding has been attained, practitioners can be trained to help implement meaningful change in the lives of individual patients (52–54).

Environment has a clear effect on health (55). Socioeconomic status has long been shown to have a causal effect on poor health (56). Marmot asserts, “The poor have poor health,” and explains that there is a social gradient in health even among those who are neither poor nor rich. He labels as “the status syndrome” the phenomenon that “The higher the social position, the better the health” (32). Once exposed to cases where this is obvious, health and social care providers understand that treating poor patients requires different behavior than their more affluent patients.

### Changing the Behavior of Global Health Patients

Providers, with adequate training, can help address the behaviors of their patients as well. It has also become evident that the environment in which people live affects their ability to make choices. Choice architecture interventions “involve altering the properties or placement of objects or stimuli within microenvironments with the intention of changing health-related behavior” (57). For example, it has become obvious that people living in material deprivation have more unhealthy cues in their environment. These include greater density of fast food, tobacco, and alcohol stores, coupled with decreased walkability of their neighborhoods (58–60). It has also been shown that cognitive capacity to resist these cues is reduced in those who were born and experienced childhood in poverty (61). They face, therefore, a “double-hit”: a reduced capacity to resist impulses created by their environment, as well as an environment that provides more cues to unhealthy behaviors (57). While free choice is preserved, the ability to exercise that choice is limited by environment.

Through a local practice, well trained professionals can help patients to educate themselves about their social determinants of health, help connect them with social support systems and healthy employment opportunities, decrease the stigma associated with disease, and focus on prevention of disease in the population. After all, “health inequalities that are preventable by reasonable means are unfair. Putting them right is a matter of social justice” (62). It has been well documented that social and behavioral interventions can make a significant impact on the health of populations that suffer from significant disparities (52–54).

Local practitioners can take an active role in using innovative methods to educate their patients about their disease processes (63). By training themselves in appropriate tools for behavior change, discussions with practitioners can have significance in altering patient behavior (54). Practitioners can successfully empower patients to make changes to behaviors that may be detrimental to their health (53). By correcting the choice architecture, local practitioners can impact individual lives (52).

### Changing the Behaviors of Society

**Figure 3:**
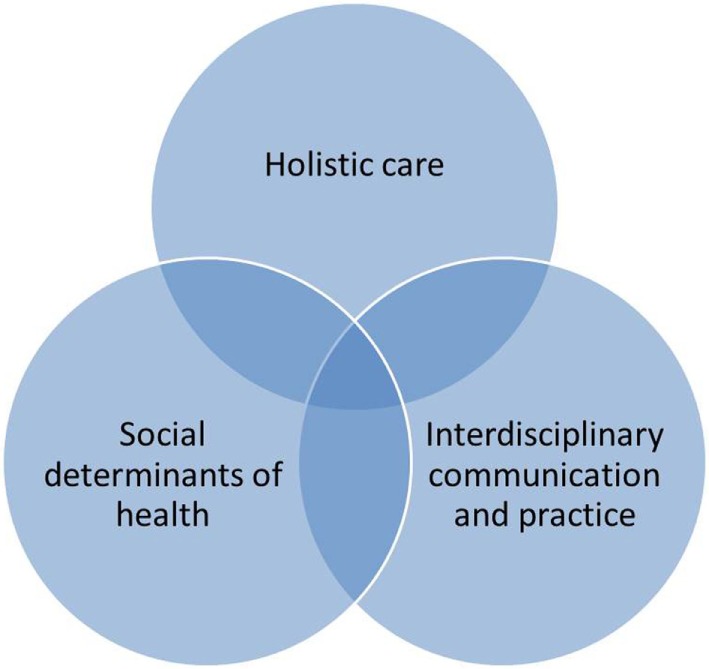
Global Health Definition.

Once providers understand the needs of individual patients and how the social determinants of health in their environment come to bear on their lives, they must advocate for a just society. “[Global health] Courses aren’t often given that orient people to the complex world and politics of international organizations” (2). As part of global health (Figure 3), providers must be trained in “systems thinking.”

[Global health education ought to involve] people who do not have [primary] expertise in health … a curriculum which makes people better able to think about how you change systems rather than just diseases, and allows them to understand much more about the system in which we’re operating. (2)

This interdisciplinary collaboration focusing on social determinants of health is the only way to guarantee holistic care for patients. As Rudolf Virchow wrote, physicians act as the, “Natural attorneys of the poor” (64). To be effective advocates, they must think appropriately regarding the issues of global health. He also wrote

Medicine is a social science and politics is nothing else but medicine on a large scale. Medicine as a social science, as the science of human beings, has the obligation to point out problems and to attempt their theoretical solution; the politician, the practical anthropologist, must find the means for their actual solution. (65)

In advocating for their patients, providers need to lobby governments at local, national and international levels as well as private corporations and NGOs. Health professionals have had great success lobbying for smoking cessation, immunizations, seat belts, and firearm violence (32, 66–68). Humanitarian diplomacy may be a formidable challenge (69). We are reminded that

The United States and United Kingdom have economies that are built on excessive consumption—including consumption of tobacco, alcohol, and food, and indeed, the use of transport powered by fossil fuels. Attempts to reduce consumption … are met with intense lobbying of governments and the public … Meeting these challenges will require vision and commitment from research funders alongside an alignment of public and political wills to implement the growing evidence base on effective interventions that could start to turn the tide on the huge and growing global burden of potentially avoidable noncommunicable diseases. (57)

It has been said that “The ‘free market’ is economic Darwinism …. By [this] reasoning, we may readily see that the poverty of the poor is justified by the richness of the rich” (70). Health professionals must advocate for a fairer society by all means available to them as this is the only way to guarantee health for our patients. This can be done in many different ways. General practice nurses and doctors will be able to discuss patient’s individual choices and help educate them in creating better lifestyles. Public health professionals can advocate for wider societal change. Researchers and editors can bring attention to social determinants of health by publishing research to fuel both patient care and advocacy.

While advocacy may seem daunting for local practitioners, it has been illustrated before in history. Thomas Hodgkin (1798–1866) was a prominent social activist as well as a clinician. As a Quaker, he vigorously opposed slavery and the slave trade and was concerned for the less fortunate in society. He worked openly, entertaining freed slaves and “educated Indians” in his home and lecturing to the working classes. This focused his concern for social inequalities in health care and he “[m]aintained that the basic problems of the poor were not medical, but socioeconomic.” He advocated for better housing for the destitute, stricter regulations on tobacco, and a public health insurance system for the poor. While this cost him advancement in his career, he remained ardent in his pursuit of justice. It is worth quoting his epitaph when describing him, as it reads “HUMANI NIHIL A SE ALIENUM PUTABAT,” or, “Nothing human was alien to him” (71). He is a member of a long legacy of health professionals advocating for social reform including Albert Schweitzer, Florence Nightingale, Rudolf Virchow as well as Bernard Lown and Yevgeni Chazov (72, 73). Each strove to attain the understanding of the social determinants of health, and once they understood, they advocated for and brought about change.

## Changing Behavior through Global Health Case Reports

The process of reading and writing global health cases can help learners understand, “the wider social, cultural, political and economic issues that may contribute to and impact … the health status of an individual” (18). In reading global health case reports, students and professionals are exposed to a multitude of determinants of health. The reports bring the weight of current medical literature to bear on individual patients and their problems. Global health case reports address the effects of poverty, ethnicity, gender, culture, stigma, conflict, refugee status, geographic location, politics, governance and other factors in the health of patients. This is the way in which medicine can, “point out problems,” as Virchow recommended. Readers can also see proposed interventions that were successful, unsuccessful or left unattempted. These are the “theoretical solutions” for which medicine ought to advocate (65). Readers are exposed to the factors which impact the health of patients around the world, and primed to recognize factors that affect health, indeed look more closely at the determinants of health of their own patients.

For example, an author may be interested in how gender affects global health. On review of BMJ Case Reports global health case reports, the reader can see the literature on how women are made vulnerable in conflict (37), the risk of culturally sanctioned sexual abuse (29), the stigma for females with sexually transmitted illness (74), disease that affects women cosmetically (75), and how poverty affects educational and economic opportunity for women (26). This informs more nuanced history taking from female patients, and more inquiry into specific determinants and risks to health. Clinicians are also moved to spend more time in educating their patients about cultural or historical factors that impede better health, and help to change the negative behaviors.

It is in the writing of global health case reports, however, that the thinking of medical professionals may be most profoundly affected. Invariably, a student or professional’s first experience of ill-health is with an individual, not a population. In their patient encounter, they will discuss the signs and symptoms of illness. However, without an analysis of the patient’s perception and these signs and symptoms and cultural understanding, opportunities to really help the patient may be missed. BMJ Case Reports has extensive guidance and a formal template for writing global health case reports and a 6-week course^2^ that students and professionals may access and use freely as they meet their patient, try to understand their perceptions of health and disease, learn more about their family, delve into the social determinants of health, explore the community, and investigate barriers to health care and health-care provision specially for their patient and then more widely for the community. Finally, after analyzing how their patient is affected, the author searches for global communities similarly affected by these global health issues. The process of getting to know the patient and his or her environment, or familiarizing oneself with world literature on global health, and finally of looking at practical, implemental solutions that bring about real change in their patient’s life is process of learning global health.

In getting to know their patients, strategies that “Rely on opportunities for person-to-person engagement and building of trust,” are necessary (47). After this, the way the patient interacts with their family and immediate society must be understood. What is the patient’s environment like at home and work? Societal standing can have profound impact on health (62). The patient’s ability to access health resources should be evaluated as well as their social capital. This may be assessed through various available tools (76). Advocacy takes place on a personal level with individual patients as well as at the societal level. As clinicians comprehend an individual’s illness and barriers to care they are better able to address their patient’s social determinants of health. Knowing more about the patient, the clinician is more invested in bringing about meaningful change in their lifestyle. Clinicians moved to write and publish a global health case report are advocating still further. It is then for editors to highlight these issues. As Hugh Clegg, former editor-in-chief of the BMJ wrote, “a subject that needs reform should be kept before the public until it demands reform” (77).

Global Health case reports also illustrate the extent to which health professionals work with families to improve health and quality of life. Richman et al. (78) report the case of a man diagnosed with a terminal brain tumor. The patient lived in a remote village and belonged to an ethnic minority—both factors limiting access to quality palliative care. The authors write

In order to facilitate care in the isolated Golan Heights village where the family home was located, two local Druze physicians set about supporting the father by training him in the variety of practices he would need to provide the services his son would require.

This effort to provide culturally and medically appropriate care was supplemented by a strong universal health-care system which included this ethnic minority, regular visits from culturally competent nurses and physicians, and strong social support. As a result of this treatment plan, the patient was able to die with the dignity appropriate to his culture and religion. The authors describe that through this intensive intervention: “[T]he family was able to provide comfort to their son, and to begin emotional healing of the family and the village in a way that could not have been accomplished far from home, in the halls of the closest hospital.” The authors finish by stating that the father now cares for another developmentally disabled son; “While the demands of care are not as involved, the father emphasizes that his capacity to care for his surviving son derives directly from his experience learning to provide comfort in the final months of his elder son’s life.”

Karande describes the case of a full-term low birth weight infant who presents with rapid weight gain over the previous 2 months (79). The mother had no antenatal or postnatal care and the delivery was conducted by a traditional birth attendant. “For complaints of ‘not growing well’ at the age of 1 month the private village doctor had started treatment with … betamethasone drops … as a ‘general tonic’.” The mother had been giving these drops for the last 3 months. The child was found on physical examination to have infantile iatrogenic Cushing syndrome (ICS) resulting from these steroid drops, and was treated by tapering off the betamethasone, and educating the mother on the discontinuation of the medication. The authors describe the failings of the rural public health system in India, which leads patients to seek care from “private non-degree allopathic practitioners (NDAPs).” Despite a lack of training in either allopathic or traditional medicine, “Most private NDAPs prescribe allopathic drugs. A large majority of NDAPs (92.7%) dispense medicines directly to patients seen by them.” The authors describe the culture of illiteracy, lack of pharmaceutical regulation, poor access to the public health-care system, and prevalence of low birthweight children conspire against infants and their mothers and make them vulnerable to risk from ICS. This infant’s health was saved by treatment and intentional education. The authors also discuss societal efforts to ensure health equity for rural and illiterate mothers and their children.

The issue of rural access to health care in India is also discussed by Shah et al. (80). A patient recently diagnosed with diabetes mellitus was given an intramuscular gluteal injection and developed injection nerve palsy. The authors sought to correct this issue by educating their staff. They

took the nursing staff in their hospital to the dissection room and demonstrated the anatomy of the sciatic nerve along with measures to prevent such injuries by demonstrating proper techniques and monitoring the staff as the practiced on cadavers.

This change in behavior and practice will help prevent future injuries. The authors also advocate for improved access to quality health care for rural residents.

A patient with HIV-associated dementia in the Dominican Republic is described by Santoso et al. (74) The patient received suboptimal treatment due to fear of stigma, a history of domestic abuse, and limited access to care. Due to the “exemplary … high quality care in a low resource setting,” this patient finally received comprehensive evaluation and treatment.

The example of professionals advocating for change in individual lives in a case describing the importance of rapport in managing chronic conditions is highlighted by Taylor and Neff (81). Further examples are in the efforts to provide treatment for a patient from a neighboring country engaged in military conflict (37), the tragic case of paired suicide in a young refugee couple facing cultural barriers in the diagnosis and detection for risk of depression and suicide among refugees on the Thai–Myanmar border (82), and a patient stigmatized by achondroplasia, facing multiple barriers to care, treated for severe hypothyroidism. The patient explains the significant change in her activities of daily living by saying, “[N]ow I feel better, it is easier to take the bus to the market because I don’t have pain in my body and no fever” (83).

On advocating for a more just practice of medicine, Kumar et al. describe the case of a woman attempting a vaginal birth after Caesarean section (VBAC) (84). Despite having had three previous Caesarean sections under the care of an obstetrician, the patient, “denied having gestational diabetes testing, blood work or detailed ultrasonography, but stated that she had undergone regular Doppler and bedside ultrasound scans performed by her midwife.” The patient chose a direct-entry midwife to provide her care, defined as

independent practitioners educated in midwifery through self-study, apprenticeship, a midwifery school or a university-based program. This is distinguished from Certified Nurse Midwives who are educated in the disciplines of nursing and midwifery, and are certified according to the requirements of the American Midwifery Certification Board. In the USA, licensure and training varies per state, with ~50% of states not requiring licensure for direct-entry midwives.

As a result of her obstetric history and the choice to seek the care of a direct-entry midwife, the patient suffered concomitant bladder and uterine rupture resulting in the loss of her pregnancy. The authors explain that many patients do not understand the difference between these two types of midwife and are left unprotected by the lack of regulation. They advocate for patient protection with more standardization and state: “It is imperative that this lack of uniformity among midwives in the USA should be addressed in the coming years.”

Jiwrajka et al. discuss the unique case of Rohingya refugees in Australia and their barriers to health care (85). The patient presented to an endocrinologist for diabetes management after being diagnosed and treated by her general practitioner. She

informed the doctors that the insulin did not ‘suit’ her and therefore had self-ceased it …. [She] had accidentally taken 600 units of … (long acting insulin glargine) over a span of 2 days resulting in near-fatal hypoglycemia. … [She] felt nauseous before losing consciousness for 30–60 minutes.

It is later discovered that, “the interpreter present at the previous consultation spoke a different dialect resulting in the misunderstanding [of correct administration and] dosage.” The patient was also concerned with her inability to conceive a child for the past 7 years, and believed “the medical profession had fixated on her diabetes,” to the neglect of her real concern, her infertility. The patient expressed her frustration by saying

I think it affects my pregnancy as well. And sometimes I think I believe in I’m not getting pregnant because of diabetes. Sometimes I just think like that. Because I don’t understand. I come from a world where people believe in medication so much, and then we think everything can be fixed with medication and I’ve been on medication for so long, and I’m not getting better, not getting fixed, like straight away.

A lack of cultural competence, inadequate translation service, and poor understanding of the patient’s primary concern of infertility conspired to put her life at risk. On realizing these issues, appropriate measures were taken to protect this patient, and the authors’ advocate for greater health equity for Rohingya refugees in Australia.

Kreniske et al. describe a case of a child wounded by a landmine to advocate for, “community landmine awareness and safety education, as well as comprehensive mine removal,” to improve health and economic viability in endemic regions (86). The case of delayed diagnosis of tuberculosis is discussed in a case report of an indigenous man in Guatemala. This is a result of poor training of rural health clinics, marginalization of indigenous people through language and cultural barriers, and inefficient health-care systems (87). The authors suggest patient accompaniment and private public partnerships to improve outcomes for these vulnerable patients. The appropriate evaluation and treatment made a profound impact on this patient as he said, “The truth is, when I went to the hospital, I was hopeless. But I thank God that it worked out. Now I am very satisfied with the treatment I received.”

### Authors and Patients as Global Health Actors

“Global Health” is becoming an increasingly prestigious field of medicine (11). Global health actors and initiatives win awards and fame as global health programmes seek funding. The *work* of global health, however, involves the holistic care of patients, real action to tackle the determinants of their health and tireless interdisciplinary communication and advocacy to work toward improving health and mitigating complications (Figure 3). The majority of actors in global health who deal daily with the complex problems of their patients and communities are those who are born, live, work, and die in the same low resource environments throughout the world as their patients. Their exhaustive work may be unreported and unsupported; most likely it will be under-funded. The patients who allow their stories to be told and the authors who champion the need for change in the social determinants of health are the “heroes” of global health. Case reports from these authors are critical to the teaching and learning of global health. Their experience in dealing every day with global health problems is invaluable to the learning of all clinicians.

## Conclusion

Global health practitioners are tomorrow’s leaders, change agents and members of effective multi-professional teams and so need to be aware of the environmental, cultural, social and political factors that impact on health, serving as advocates of people’s rights to access resources, education and healthcare. (88)

As McKimm and McLean go on to explain “institutions should be producing graduates who can think globally but act locally to deliver appropriate health care and adapt to the changing needs of communities and populations, irrespective of where they practice medicine.” Even if a health professional never leaves his hometown, he will be practising global health, as his treatment of the patient will involve a focus on the determinants of health and disease as well as the immediate management of pathology. These professionals must be able to assess the social determinants of health to guarantee health equity for their patients. Public health professionals can advocate for societal change to improve the choice architecture of populations. Researchers are encouraged to publish papers that keep these issues in the public eye until the public demands change. BMJ Case Reports global health case reports serve to expand the evidence base to fight for change in the determinants that adversely affect health and access to quality health care.

There is a need to develop a repository that documents the determinants of health as they affect real patients, to use this evidence base in advocating for better health and better health care for individuals and societies and to take on global health problems at the level of the individual. Studying global health issues at the level of individual patients brings home the realities of complex global health problems that may seem too daunting to take on. Tackling the determinants of health one patient at a time, though, gives clinicians real hope in achieving global health goals. Exposure to this literature primes clinicians to think of the social determinants of health, not merely the biochemical pathology of disease. This change in how we as clinicians think about patients and illness is essential if we are to address the underlying causes of illness and not return patients to the same environments that made them ill. Clinicians, in turn, must use their understanding of the determinants of health to implement culturally competent and effective behavioral change strategies for their patients as individuals. Together, through global health case reports, patients, their medical teams and editors must advocate locally, nationally and globally for meaningful change in the social determinants of health. This is a global responsibility.

## Author Contributions

ND and SB both made substantial contributions to the conception or design of the work, were involved in drafting the work or revising it critically for important intellectual content, gave final approval of the version to be published, and agreed to be accountable for all aspects of the work in ensuring that questions related to the accuracy or integrity of any part of the work are appropriately investigated and resolved.

## Conflict of Interest Statement

SB is a paid editor-in-chief of BMJ Case Reports. The authors declare that the research was conducted in the absence of any commercial or financial relationships that could be construed as a potential conflict of interest.

## Footnotes

^1^http://casereports.bmj.com/search?submit=yes&collection_num=2263. (Accessed: March 1, 2017).

^2^http://casereports.bmj.com/site/misc/GHMA_Mar_2017.pptx. (Accessed: March 1, 2017).
